# Heterozygous caveolin‐3 mice show increased susceptibility to palmitate‐induced insulin resistance

**DOI:** 10.14814/phy2.12736

**Published:** 2016-03-31

**Authors:** M. A. Hassan Talukder, Marilena Preda, Larisa Ryzhova, Igor Prudovsky, Ilka M. Pinz

**Affiliations:** ^1^ Maine Medical Center Research InstituteScarboroughMaine

**Keywords:** Caveolae, caveolin, CD36, heart, insulin resistance, lipid

## Abstract

Insulin resistance and diabetes are comorbidities of obesity and affect one in 10 adults in the United States. Despite the high prevalence, the mechanisms of cardiac insulin resistance in obesity are still unclear. We test the hypothesis that the insulin receptor localizes to caveolae and is regulated through binding to caveolin‐3 (CAV3). We further test whether haploinsufficiency for CAV3 increases the susceptibility to high‐fat‐induced insulin resistance. We used in vivo and in vitro studies to determine the effect of palmitate exposure on global insulin resistance, contractile performance of the heart in vivo, glucose uptake in the heart, and on cellular signaling downstream of the IR. We show that haploinsufficiency for CAV3 increases susceptibility to palmitate‐induced global insulin resistance and causes cardiomyopathy. On the basis of fluorescence energy transfer (FRET) experiments, we show that CAV3 and IR directly interact in cardiomyocytes. Palmitate impairs insulin signaling by a decrease in insulin‐stimulated phosphorylation of Akt that corresponds to an 87% decrease in insulin‐stimulated glucose uptake in HL‐1 cardiomyocytes. Despite loss of Akt phosphorylation and lower glucose uptake, palmitate increased insulin‐independent serine phosphorylation of IRS‐1 by 35%. In addition, we found lipid induced downregulation of CD36, the fatty acid transporter associated with caveolae. This may explain the problem the diabetic heart is facing with the simultaneous impairment of glucose uptake and lipid transport. Thus, these findings suggest that loss of CAV3 interferes with downstream insulin signaling and lipid uptake, implicating CAV3 as a regulator of the IR and regulator of lipid uptake in the heart.

## Introduction

Insulin resistance and type II diabetes are comorbidities of obesity, which has reached epidemic proportions in the United States. One in 10 adults has diabetes in the United States and this ratio is projected to rise to one third of adults by 2050 (CDC, 2010). The incidence of heart disease including diabetic cardiomyopathy with subsequent heart failure accounted for 62% of all diabetes‐related deaths in those aged 65 and older in the United States in 2007 (CDC, National Diabetes Fact Sheet, 2010). Therefore, there is a clear link between obesity, insulin resistance, and heart failure, although causal mechanisms are not clearly defined. Our laboratory showed that mice fed a high‐fat (palmitate) diet for 12 weeks exhibit the onset of insulin resistance and systolic contractile dysfunction. These results support the concept that a palmitate‐rich diet induces early changes in cardiac metabolism and function, particularly dysregulation of the insulin receptor cascade.

### Caveolae and caveolins

Caveolae are membrane invaginations rich in ceramide, sphingomyelin, cholesterol, and membrane proteins called caveolins (Anderson 1998; Smart et al. 1999). Caveolae regulate endocytosis, lipid trafficking, and signal transduction (Okamoto et al. 1998) in cells including fibroblasts, adipocytes, endothelial cells, and skeletal and cardiac myocytes (Severs 1988). There are three caveolin isoforms: caveolin‐1 (CAV1), caveolin‐2 (CAV2), and caveolin‐3 (CAV3). While CAV1 is ubiquitously expressed, CAV3 is expressed exclusively in skeletal and cardiac muscle (Li et al. 1995; Tang et al. 1996; Scherer et al. 1997). Caveolins bind to cholesterol, localize along the inner leaflet of the plasma membrane forming caveolae (Monier et al. 1996; Stan 2005), and act as scaffolding proteins that maintain caveolae structure. Loss of CAV3 results in the absence of caveolae in cardiomyocytes and induces hypertrophy in the heart (Galbiati et al. 2001; Park et al. 2002; Woodman et al. 2002; Augustus et al. 2008). Signaling proteins such as the IR (Gustavsson et al. 1999), H‐Ras (Song et al. 1996), G proteins and G‐protein‐coupled receptors, and nitric oxide synthase (Feron et al. 1996) are found in caveolae. CAV1 and 3 can bind with and regulate the activity of these signaling proteins via their caveolin‐scaffolding domain (CSD) (Couet et al. 1997a,b).

### Regulation of IR signaling by caveolins

The IR binds to the CSD of caveolins through its caveolin‐binding motif, located within the kinase domain of the beta‐subunit. Caveolins regulate IR activity through this interaction (Yamamoto et al. 1998). Furthermore, mutations within the caveolin‐binding motif affect IR expression and autophosphorylation (Nystrom et al. 1999). Such IR mutations within the caveolin‐binding motif have been identified in patients with inherited severe insulin resistance and type II diabetes (Moller et al. 1990; Nystrom et al. 1999; Imamura et al. 2001). Global CAV3 null mice are reported to have a distinct phenotype with increased body mass, insulin resistance, hyperinsulinemia, and impaired glucose tolerance (IGT) compared to littermate wild‐type mice (Woodman et al. 2002; Oshikawa et al. 2004; Capozza 2005), and they develop cardiomyopathy at 8 weeks of age. These findings implicate caveolins in IR activity, stability, and localization to caveolae in skeletal muscle. Although the CAV3 and IR interaction and the effects of loss of CAV3 on insulin signaling have been characterized in skeletal muscle (Oshikawa et al. 2004), the role of CAV3 in insulin signaling in cardiomyocytes is poorly understood. We chose to use CAV3^+/−^ mice because under normal baseline conditions they do not present a skeletal or cardiac muscle phenotype; thus, CAV3^+/−^ mice are a good experimental model to characterize their susceptibility to lipid‐induced insulin resistance and cardiac dysfunction.

### Palmitate and lipid membrane composition in insulin resistance

Plasma membrane lipid composition is important for maintaining caveolae structure and function. Cholesterol depletion induces loss of caveolae invagination and disrupts insulin signaling in adipocytes (Parpal et al. 2001). In addition, ceramide has been shown to compete with and displace cholesterol in synthetic unilamellar membrane systems (Megha, 2004). Therefore, increased de novo synthesis and accumulation of ceramide, driven by the exogenous saturated fatty acid, palmitate, may be sufficient to impair signal transduction of IR by disrupting lipid membrane integrity and caveolae structure. We have shown in the mouse that downregulation of caveolins in the heart is a lipid species‐dependent process (Knowles et al. 2011, 2013). A high‐palmitate diet is sufficient to increase cardiac ceramide and sphingomyelin levels and decrease CAV3 expression and plasma membrane localization in the heart (Knowles et al. 2011, 2013). This project will test the novel concept that a high‐palmitate diet disrupts caveolae function and thus contributes to impaired insulin signaling and insulin resistance through loss of IR regulation by CAV3.

The increasing prevalence of obesity and type II diabetes makes it imperative to determine the mechanisms of such associated morbidities, which contribute to heart disease. The consequences of lipid‐induced loss of important structural and regulatory proteins such as caveolins and how such changes affect IR activity in the heart is poorly understood and is thus the focus of this work as a novel mechanism of high‐fat‐induced insulin resistance.

## Material and Methods

### Animals

Caveolin‐3 (CAV3) mice were generated by using ES cells (CAV3^tm1(KOMP)Vlcg^) on a C57BL/6NTac genetic background obtained from the KOMP repository at UC Davis. The ES cells were injected into C57BL/6J morulae and implanted into pseudopregnant FvB mice. The CAV3 mouse colony was maintained as a heterozygous breeding colony by crossing heterozygous siblings. Male and female wild‐type (CAV3^+/+^) and littermate heterozygous (CAV3^+/−^) mice were used throughout the experiments. Genotyping was performed using a wild‐type CAV3 forward (CTTGGGTTATCCAGCCAGTAAG), a wild‐type CAV3 reverse (GGGTTTTAACTGCACCAGGGATCAC), and a forward primer detecting the 3′ end of the neocassette insertion (GCAGCCTCTGTTCCACATACACTTCATTCT) with normal PCR settings and 35 cycles. The Maine Medical Center Institutional Animal Care and Use Committee approved all experimental protocols. The handling and housing of mice followed the recommendations of current NIH and American Physiological Society guidelines for the care and use of laboratory animals.

### Diet composition and feeding regimen

The high‐fat feeding regimen was established previously (Knowles et al. 2013) and repeated for these studies. Briefly, high‐lipid diets were custom‐made by Harlan Laboratories and all high‐fat diets contained 200 g/kg fat (20%). In the high‐palmitate diet (Harlan TD05235), 41% of all fat was palmitate and termed as PALM diet. As control for the PALM diet, a diet was composed of medium chain triglycerides with the same caloric intake from fat (Harlan TD05237) and termed as MCT diet. Standard laboratory chow had a crude fat content of 6% and termed as STD diet. Wild‐type (CAV3^+/+^) and littermate heterozygous (CAV3^+/−^) mice of both genders (*n* = 6/gender/diet) were randomly assigned to high‐fat diets at the age of 3–4 weeks and maintained for 12 weeks. Body weight (BW) was monitored weekly from the start of diet regimen. From 6 weeks on, mice underwent echocardiography every 2 weeks. Glucose tolerance testing was performed at 6 and 12 weeks. Hearts and skeletal muscle from mice were harvested at 12 weeks, flash frozen, and used for the determination of protein expressions by western blot.

### Glucose tolerance test (GTT)

Food was removed in the morning and the experiments were performed in the afternoon after 6 h of fasting. Blood glucose was measured at 0, 15, 30, 60, and 120 min after i.p. injection of 50% sterile glucose solution at a dose of 1.5 g/kg. Blood glucose was measured by sampling blood from the tail tip with a glucometer.

### Echocardiography

Mice underwent biweekly echocardiography for morphological and functional assessment of the heart starting at 6 weeks until 12 weeks. Mice were anesthetized with isoflurane (1.0–1.5%) and placed supine on a heated stage. Paws were taped to ECG leads and the fur removed from the chest area using Nair hair removal. Echocardiography was performed with a Vevo 2100 (VisualSonics Systems, Toronto, ON, Canada), 40 MHz solid‐state transducer, and the papillary muscle was used as orientation for the acquisition of short‐axis M‐mode images. Data were analyzed by the heart function package provided by VisualSonics Systems.

### Cell culture

The HL‐1 cell line is a cardiomyocyte line derived from the AT‐1 mouse atrial cardiomyocyte lineage, established and described by Claycomb (Moller et al. 1990). Culture of cells followed the procedure established by Calycomb et al. (Claycomb et al. 1998). Cultures were grown to 75% confluency before treatment and 100% confluency before being passaged or harvested for analysis. Experimental treatment of the cells was started after 24 h of culture (30–50% confluency) in supplemented Claycomb's medium. The cells were then washed with serum‐free Claycomb's medium, and cultured in the medium for 12–24 h prior to treatment with experimental conditions. To simulate the Claycomb's medium, while maintaining control of BSA‐bound palmitate, the following components were added to Dulbecco's modified Eagle's minimal essential media (DMEM) creating a basal serum‐free medium: 2 mg/mL BSA, 2 mmol/L l‐carnitine, 5 mmol/L creatine, 5 mmol/L taurine, 1 mmol/L l‐glutamine, 100 units/mL penicillin, 100 *μ*g/mL streptomycin, 0.1 mmol/L norepinephrine (added fresh at the day of use). Control medium comprised basal medium supplemented with 5% fatty acid‐free BS, whereas experimental medium contained 0.4 mmol/L palmitate bound to 5% fatty acid‐free BSA. Cells were exposed to 0.4 mmol/L palmitate medium for 18 h. Myriocin treatment was performed by dissolving a 1‐mmol/L stock solution to the effective dose of 40 nmol/L in each culture dish.

### SiRNA transfection

CAV3 knockdown in HL‐1 cells was performed using cav3 siRNA (Santa Cruz) and lipofectamine 2000 (Invitrogen) as per manufacturer's protocol. A scrambled nontargeting siRNA was used as control (Santa Cruz). Prior to transfection, HL‐1 cells were placed in antibiotic‐free growth medium. At 30–50% confluency, 100 pmol siRNA were added to each well and incubated at 37°C for 20 h. Verification of Cav3 knockdown was determined by western blotting.

### Glucose uptake assay

Primary isolated cardiomyocytes and HL‐1 cells were tested for glucose uptake using essentially the same method. HL‐1 cells were maintained in serum‐free medium for 18 h and primary cardiomyocytes were used fresh. Cells were incubated in HEPES‐buffered saline solution (140 mmol/L NaCl, 5 mmol/L KCl, 2.5 mmol/L MgSO_4_, 1 mmol/L CaCl_2_, 20 mmol/L HEPES, pH 7.4) for 30 min to deplete intracellular glucose, followed by incubation in 0.1 mmol/L 2‐deoxy‐d‐glucose and 1 *μ*Ci/mL 2‐deoxy‐d‐[^3^H]‐glucose with and without 200 nmol/L insulin for 10 min. Cells were solubilized in 0.5% Triton X. Radioactivity of uptaken [^3^H] 2‐deoxyglucose was determined by a scintillation counter in counts/min and total glucose uptake was calculated as pmol/mg protein/min.

### Isolation of adult mouse cardiomyocytes

Adult mouse cardiomyocytes were isolated following Pinz et al. (Pinz et al. 2011). Cardiomyocytes were readjusted to 1.2 mmol/L extracellular calcium and plated on mouse laminin‐coated coverslips. Freshly isolated cells were used for glucose uptake measurements or fixed the same day for confocal microscopy.

### Confocal microscopy

After adhesion to laminin‐coated coverslips, cardiomyocytes were washed twice in ice‐cold PBS and fixed with 4% paraformaldehyde in PBS for 10 min at room temperature. Coverslips were washed with ice‐cold PBS and stored in PBS with 0.01% sodium azide. Fixed cells were blocked and permeabilized with 5% BSA, 0.1% Triton‐X‐100, 0.1% Tween20, and 0.1% sodium azide in PBS for 1 h at room temperature. Primary antibodies were diluted in PBS with 1% BSA and 0.3% Triton‐X‐100: Caveolin‐3 (BD Transduction #610420, 1:300), p‐IRS1 (Cell Signaling #2381 Ser307, 1:100), and Akt (Cell Signaling, 1:500). Fixed cells were incubated with primary antibodies at 4°C overnight. Next, cells were washed in PBS and incubated for 2 h with Alexa Fluor 488‐labeled goat anti‐mouse IgG 1:2000 (Invitrogen) and Alexa Fluor 546 goat anti‐rabbit IgG 1:2000 (Invitrogen). Counterstaining with a DNA stain TOPRO (Invitrogen T3605, 1:1000) was completed for 30 min at room temperature directly after secondary antibody incubation. Cells were embedded in Gold antifade mounting media (Invitrogen) and examined with a Leica TCS SP1 or SP8 Confocal Laser Scanning Microscope and analyzed using the Leica confocal software. For FRET experiments in fixed cells, primary antibodies were directly labeled by Alexa Fluor 488 or Alexa Fluor 555 using APEX Antibody Labeling Kits (Molecular Probes). In FRET studies, fluorescence recovery after photobleaching approach was used. Alexa 555 fluorescence in individual cells was specifically bleached, and the intensities of Alexa 488 images before and after bleaching were compared. At least 10 cells per experiment were investigated.

### Western blot analysis

Western blotting was performed to determine the relative expression of cardiac and skeletal muscle proteins following 12 weeks of diet regimen. Briefly, frozen heart and skeletal muscle samples were homogenized in ice‐cold lysis buffer containing HEPES (20 mmol/L), NaCl (150 mmol/L), MgCl_2_ (1.5 mmol/L), glycerol (10%), Triton‐X 100 (1%), EDTA (1 mmol/L), protease‐inhibitor cocktail (Calbiochem), and PhosSTOP Phosphatase Inhibitor Cocktail Tablets (Roche), and centrifuged at 15.7 *g* for 30 min at 4°C. Protein concentration of the supernatant was determined according to Lowry et al. (Lowry et al. 1951). The homogenate was mixed with the sample loading buffer at a ratio of 1:1 (v/v) and boiled for 5 min. Equal amounts of sample proteins (50 *μ*g) were subjected to SDS‐PAGE and transferred to nitrocellulose membranes at room temperature. Membranes were blocked for 2 h at room temperature in Tris‐buffered saline (TBS) containing 0.05% Tween‐20 (TBS‐T) and 5% nonfat dry milk. Membranes were then incubated overnight at 4°C with primary antibodies (source; dilution) against the following proteins: caveolin‐3 (BD Transduction Laboratories; 1:2000), caveolin‐1 (Cell Signaling; 1:1000), insulin receptor *β* (Cell Signaling; 1:1000), GLUT4 (Santa Cruz; 1:100), CD36 (GeneTex; 1:500), and GAPDH (Santa Cruz; 1:3000). After overnight incubation, the membranes were washed three times (10 min each) in TBS‐T, and incubated for 1 h with horseradish peroxidase‐conjugated anti‐rabbit or anti‐mouse IgG in TBS‐T with 5% nonfat dry milk at room temperature. Membranes were then washed three times in TBS‐T, and immunoreactive bands were visualized using FUJIFILM LAS‐4000 Luminescent Image Analyzer and chemiluminescent HRP antibody detection substrate Luminata Forte (Millipore). The signal intensity of scanned blotting was analyzed using NIH ImageJ software and GAPDH was used as an internal control for equal protein loading.

### Data analysis

All results are expressed as the mean ± SEM. Data were analyzed either by two‐tailed Student's *t* test for paired data from the same experiment and unpaired data from different experiments or by ANOVA followed by Student–Newman–Keul's post hoc test or by two‐way ANOVA followed by Bonferroni–Dunn comparison. Values of *P* < 0.05 were considered to be significant.

## Results

### General characteristics of mice and response to high‐fat diets

**Figure 1:**
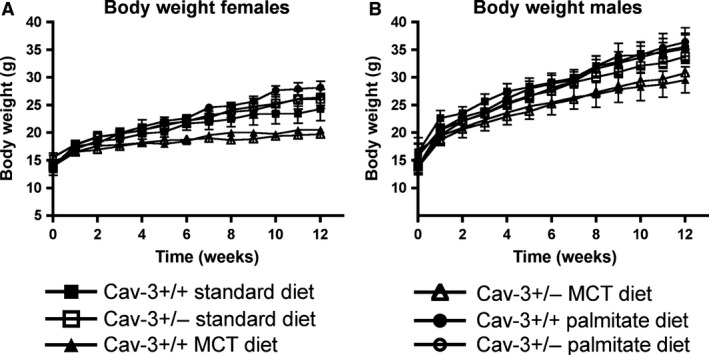
Body weights and heart chamber weights of CAV3^+/+^ and CAV3^+/−^ during 12 weeks of the diet regimen. (A, B) There is no difference between body weight gain in CAV3^+/+^ and CAV3^+/−^ fed standard laboratory chow, MCT, or palmitate diet. In both genders (A, females; B, males) the MCT diet caused a slight, not significant, weight decrease. Data are means ± SE,* n* = 7–12.

To assess the role of CAV3 in the palmitate‐induced susceptibility to insulin resistance, CAV3^+/−^ and control CAV3^+/+^ 3 weeks old mice were transferred for 12 weeks to the following diets: (1) high‐caloric palmitate‐rich diet (PALM); (2) control low‐caloric (STD) diet; and (3) control high‐caloric diet where palmitate is replaced by triglycerides with medium length fatty acid residues (MCT). Gross phenotypic changes in CAV3^+/+^ and CAV3^+/−^ mice maintained at each diet were determined by weekly measuring body weight (BW) gain and by determining heart weight (HW) after 12 weeks of feeding. Figure 1 shows that male mice had accelerated BW gain compared to female mice with all diets, and mice maintained on STD or PALM diet had slightly higher weight gain compared to MCT diet. Longitudinal evaluation and quantitation of these findings, however, demonstrated no significant differences in BW gain between CAV3^+/+^ and CAV3^+/−^ mice with any diet (Fig. 1 and Table 1). HW and BW/HW ratios, which are the gross measure of hypertrophic changes in the heart, were unchanged in all groups (Table 1). Thus, there were no morphological differences caused by the high‐fat diets used in this study.

**Table 1:** Body and heart weights of CAV3^+/+^ and CAV3^+/−^ mice after 12 weeks of the diet regimen

	CAV3^+/+^ standard	CAV3^+/−^ standard	CAV3^+/+^ MCT	CAV3^+/−^ MCT	CAV3^+/+^ palmitate	CAV3^+/−^ palmitate
*N* and gender	8 (4 m, 4f)	10 (4 m, 6f)	7 (5 m, 2f)	10 (6 m, 4f)	8 (4 m, 4f)	12 (7 m, 5f)
BW (end of diet)	32.3 ± 2.9	31.2 ± 1.9	27.4 ± 2.2	28.0 ± 1.6	34.6 ± 1.8	31.4 ± 1.9
Atria (mg)	5.5 ± 0.3	5.9 ± 0.3	6.3 ± 0.6	6.1 ± 0.2	6.5 ± 0.3	5.6 ± 0.3
Right ventricle (mg)	18.0 ± 1.6	17.5 ± 1.2	18.0 ± 1.5	17.4 ± 0.9	17.8 ± 1.0	19.5 ± 1.4
Left ventricle (mg)	91.3 ± 5.8	84.5 ± 3.9	86.0 ± 4.7	84.4 ± 3.5	87.6 ± 4.0	87.3 ± 4.4
LV volume (*µ*L)	15.5 ± 1.1	13.8 ± 0.7	14.9 ± 1.0	14.0 ± 0.7	13.4 ± 0.6	14.3 ± 0.6
Heart weight (mg)	115 ± 7	108 ± 5	110 ± 6	108 ± 4	112 ± 4	112 ± 5
BW/HW (g/mg)	3.6 ± 0.1	3.5 ± 0.1	4.1 ± 0.3	3.9 ± 0.1	3.3 ± 0.1	3.7 ± 0.2

BW, body weight; HW, heart weight; LV, left ventricle.

### Diminished in vivo cardiac performance in CAV3^+/−^ mice with high‐fat diets

**Figure 2:**
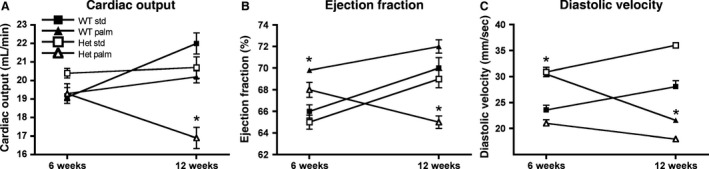
Echocardiography at 6 and 12 weeks of standard or palmitate diet feeding. At the 12‐week time point CAV3^+/−^ mice fed palmitate diet have lower cardiac output (A), lower ejection fraction (B), and lower diastolic velocity (C) compared to CAV3^+/+^ mice. Data are means ± SE,* n* = 5–6, *P < 0.05 versus WT standard diet, ^#^ *P *< 0.001 versus all other groups, two‐way ANOVA with Bonferroni–Dunn post hoc comparison.

Previously, we have reported that PALM diet causes systolic and diastolic dysfunction in the isolated Langendorff heart (Knowles et al. 2013). To investigate if PALM diet feeding has any effect on the heart in vivo, we performed echocardiography on mice starting from 6 weeks of age every 2 weeks in all diet‐fed mice. We measured the function of the heart as ejection fraction (EF) and fractional shortening (FS) as well as determined left ventricular (LV) volumes, wall thickness, and dimensions during systole and diastole. The results (Table 2) showed no significant differences in these parameters between STD diet‐fed CAV3^+/+^ and CAV3^+/−^ mice at baseline and at 12 weeks of age. Data for mice fed MCT diet are not shown, because they are indistinguishable from STD diet. After 12 weeks of PALM diet feeding we observed modest changes in the CAV3^+/+^ mice with lower cardiac output but preserved ejection fraction compared to STD‐fed mice. Diastolic velocity decreased significantly in CAV3^+/+^ hearts suggesting that in vivo PALM feeding leads to diastolic changes preceding systolic changes. This is in contrast to our previous findings in the ex vivo Langendorff heart, where wild‐type C57BL6 mice after 12 weeks of PALM feeding showed severe systolic and diastolic dysfunction (Knowles et al. 2013). This finding does not contradict our previous results, because in vivo the heart has humoral and neuronal support that can compensate a decline in contractile function. In the CAV3^+/−^ mice, however, cardiac output, ejection fraction, and diastolic velocity were affected (Fig. 2B–D), suggesting that on the heterozygous background mice are more susceptible to PALM diet and that compensatory mechanisms fail to preserve contractile function in vivo.

**Table 2:** Echocardiography at 6 and 12 weeks of the diet regimen in CAV3^+/+^ and CAV3^+/−^ mice

	CAV3^+/+^				CAV3^+/−^			
	6 weeks		12 weeks		6 weeks		12 weeks	
	Standard	Palmitate	Standard	Palmitate	Standard	Palmitate	Standard	Palmitate
Genders	2f, 3 m	3f, 3 m	2f, 3 m	3f, 3 m	3f, 3 m	3f, 3 m	3f, 2 m	3f, 2 m
IVS;d (mm)	0.74 ± 0.03	0.81 ± 0.03	0.84 ± 0.03	0.82 ± 0.01	0.77 ± 0.05	0.74 ± 0.01	0.80 ± 0.02	0.78 ± 0.02
IVS;s (mm)	1.12 ± 0.03	1.09 ± 0.03	1.20 ± 0.04	1.20 ± 0.07	1.00 ± 0.03	0.99 ± 0.03	1.14 ± 0.05	1.11 ± 0.04
LVID;d (mm)	3.71 ± 0.04	3.57 ± 0.05	3.88 ± 0.13	3.61 ± 0.07	3.85 ± 0.13	3.77 ± 0.13	3.78 ± 0.17	3.71 ± 0.03
LVID;s (mm)	2.40 ± 0.05	2.10 ± 0.04	2.38 ± 0.13	2.15 ± 0.09	2.54 ± 0.14	2.40 ± 0.11	2.37 ± 0.15	2.35 ± 0.06
LVPW;d (mm)	0.74 ± 0.02	0.71 ± 0.05	0.81 ± 0.06	0.80 ± 0.07	0.72 ± 0.03	0.75 ± 0.02	0.81 ± 0.05	0.74 ± 0.04
LVPW;s (mm)	1.09 ± 0.05	1.16 ± 0.06	1.23 ± 0.04	1.23 ± 0.08	1.03 ± 0.05	1.14 ± 0.04	1.17 ± 0.07	1.15 ± 0.03
Volume;d (*µ*L)	58.8 ± 1.7	52.8 ± 2.3	62.6 ± 6.3	55.8 ± 2.3	64.4 ± 5.4	58.2 ± 2.2	55.8 ± 3.6	57.7 ± 1.5
Volume;s (*µ*L)	19.1 ± 1.4	15.3 ± 0.6	20.2 ± 2.6	15.6 ± 1.4	21.7 ± 2.5	18.1 ± 1.3	17.2 ± 1.8	20.1 ± 1.0
Cardiac Output (mL/min)	19.1 ± 0.4	19.3 ± 0.8	22.0 ± 1.4	20.2 ± 0.8*	20.4 ± 0.6	19.3 ± 1.3	20.7 ± 1.4	16.9 ± 1.4*
Ejection fraction (%)	66 ± 1.5	68.9 ± 0.5	70 ± 2.4	72 ± 1.5	65 ± 1.6	68 ± 1.7	69 ± 2.0	65 ± 1.4*
Fractional shortening (%)	36.0 ± 1.1	40.8 ± 1.5	39.2 ± 1.9	40.8 ± 1.3	34.9 ± 1.1	38.1 ± 1.1	37.7 ± 1.6	35.1 ± 1.1
Heart rate (bpm)	478 ± 35	507 ± 14.2	486 ± 11	501 ± 14.2	516 ± 19	482 ± 36	516 ± 15	476 ± 23
Stroke volume (*µ*L)	38.5 ± 1.3	38.3 ± 2.1	45.6 ± 3.6	40.2 ± 1.1	41.3 ± 2.7	40.1 ± 1.5	38.3 ± 2.8	37.6 ± 1.3
Diastolic velocity (mm/sec)	23.6 ± 2.2	30.5 ± 1.5*	28.1 ± 2.8	21.6 ± 1.1*	30.9 ± 2.2#	21.2 ± 1.7#, *	36.0 ± 1.2#	18.8 ± 1.2*
Diastolic time (msec)	50.7 ± 2.6	49.7 ± 1.1	53.6 ± 1.8	49.8 ± 2.5	47.9 ± 2.2	50.0 ± 3.2	46.8 ± 1.1	59.2 ± 4.3*
Systolic velocity (mm/sec)	20.0 ± 0.9	21.3 ± 1.0	25.8 ± 1.3	19.9 ± 2.6	19.8 ± 1.5	22.1 ± 2.1	21.1 ± 1.2	19.0 ± 1.6
Systolic time (msec)	44.6 ± 2.5	41.3 ± 0.7	44.0 ± 1.0	44.7 ± 0.9	43.3 ± 1.1	43.7 ± 2.8	43.6 ± 1.7	43.6 ± 2.9

IVS, interventricular septum; LVID, left ventricular inner diameter; LVPW, left ventricular posterior wall; d, diastolic; s, systolic.

*N* = 5–6, *P, 0.05 versus standard diet in group, ^#^ *P*, 0.05 versus respective diet between groups, two‐way ANOVA, Bonferroni–Dunn post hoc comparison.

### Accelerated onset of glucose intolerance in CAV3^+/−^ mice maintained on palmitate‐rich diet

**Figure 3:**
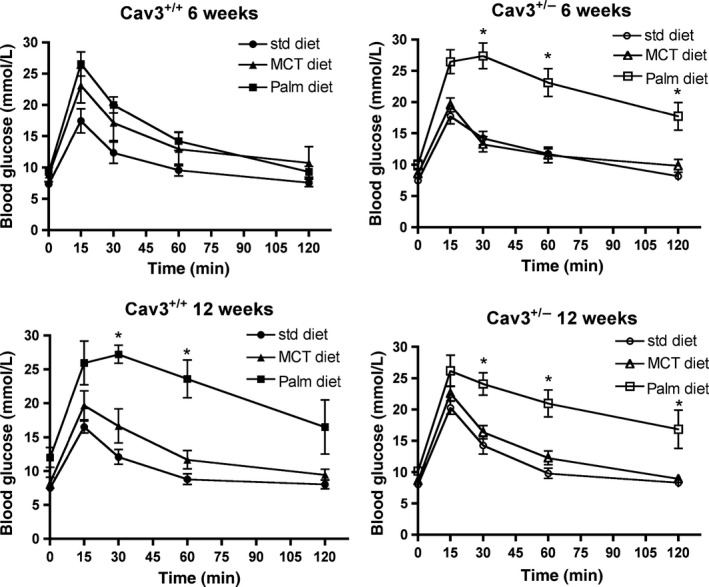
Glucose tolerance testing in CAV3^+/+^ and CAV3^+/−^ mice at 6 and 12 weeks of high‐fat diets. CAV3^+/−^ and their littermate CAV3^+/+^ mice were fed standard chow, MCT, or palmitate diet for 12 weeks. Glucose tolerance was determined after 6 and 12 weeks by i.p. injection of 1.5 g/kg of glucose solution. Data show that CAV3^+/−^ mice are more susceptible to palmitate‐induced glucose intolerance. Only palmitate‐fed CAV3^+/−^ mice show glucose intolerance at 6 weeks. By 12 weeks, all palmitate diet‐fed groups are glucose intolerant *P < 0.01, repeated measures ANOVA.

Loss of CAV3 has been reported to cause impaired lipid metabolism and insulin resistance in mice (Galbiati et al. 2001; Capozza 2005). We have recently demonstrated that feeding high‐palmitate diet in mice can induce a loss of cell membrane CAV3 (Knowles et al. 2011, 2013). Hyperlipidemia is an important cause of glucose intolerance in humans; therefore, we were interested to know how high‐fat diets can influence glucose tolerance in CAV3^+/−^ mice. We determined glucose tolerance in all groups of mice at 6 weeks and 12 weeks of feeding regimens. While glucose tolerance in CAV3^+/+^ mice was comparable between all diet groups at the 6‐week point (Fig. 3A), CAV3^+/−^ mice with PALM diet displayed significantly elevated blood glucose levels at 15‐min post glucose load (Fig. 3B), which remained elevated for 120 min as compared to STD and MCT groups. Interestingly, at 12‐week point, in addition to persistently impaired glucose tolerance in CAV3^+/−^ mice (Fig. 3D), CAV3^+/+^ mice with PALM diet also displayed significantly elevated blood glucose levels at 30 and 60 min (Fig. 3C) compared to STD and MCT groups. Thus, CAV3^+/−^ mice with PALM diet were more susceptible to glucose intolerance with an accelerated onset than CAV3^+/+^ mice indicating an important role of CAV3 in blood glucose regulation in a high dietary palmitate environment.

### Insulin receptor (IR) and CAV3 colocalize and directly interact in HL‐1 cell plasma membrane

**Figure 4:**
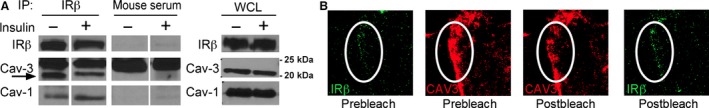
IR interacts with CAV1 and CAV3 in cardiomyocytes. (A) IR was immunoprecipitated (IP) with an antibody against the beta chain of IR (IR *β*) from HL‐1 cells challenged with or without 200 nmol/L insulin for 5 min, and immunoblotted for CAV1, CAV3, and the IR *β* subunit. The strong band above CAV3 is IgG, which is recognized by the anti‐mouse antibody. Mouse serum was used as immunoprecipitation control. IR, CAV1, and CAV3 were detected in whole cell lysates (WCL) from each pre‐IP sample. (B) Representative FRET images in fixed HL‐1 cells demonstrated colocalization of CAV3 and IR. After acceptor bleaching (white circle) there was a 50% increase in donor fluorescence (postbleach IR *β* image). Both experiments suggest that CAV1 and CAV3 are in close proximity to the IR. FRET repeated in four different experiments with similar results.

IR signaling in skeletal muscle of CAV3 null mice is reported to be dependent on CAV3 expression; however, the direct interaction of caveolins with IR remains controversial. With growing evidence for a role of CAV3 in IR signaling (Yamamoto et al. 1998; Oshikawa et al. 2004; Otsu et al. 2010), we performed experiments to determine if IR and CAV3 directly interact. Coimmunoprecipitation experiments in HL‐1 cells show that IR forms complexes with CAV3 and CAV1 in HL‐1 in the presence and absence of insulin (Fig. 4A; left panel). To further support our conclusion that IR and CAV3 interact in cardiomyocytes, we performed fluorescence energy transfer (FRET) experiments in fixed HL‐1 cells, to exclude any lateral mobility of proteins in the plasma membrane. To that end, CAV3 was detected using Alexa 555‐conjugated antibodies, and IR*β* using Alexa 488‐conjugated antibodies. Figure 4B shows representative FRET images in HL‐1 cells indicating the direct interaction of CAV3 and IR. After Alexa 555 acceptor bleaching (bleached area in white circle) there was a 37 ± 11% increase in Alexa 488 donor fluorescence (postbleach IR*β* image minus prebleach IR*β* image). These results suggest that CAV3 and IR are in close proximity in HL‐1 cell membrane and most likely bind directly to each other (Fig. 4B), and this interaction may play important roles in the process of IR activation and subsequent signaling.

### Loss of CAV3 in HL‐1 cells and primary cardiomyocytes abolishes insulin stimulated glucose uptake

**Figure 5:**
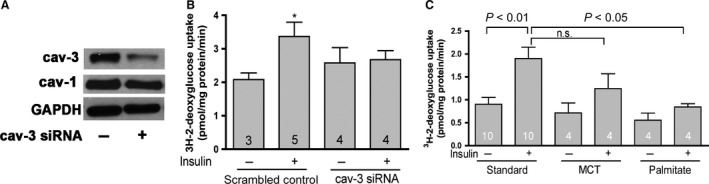
Loss of CAV3 abolishes insulin sensitivity in HL‐1 cells and in primary isolated cardiomyocytes. (A) HL‐1 cells were transfected with 100 pmol of *CAV3* siRNA using lipofectamine 2000 and collected for western blotting 20 h after transfection. CAV3 protein is decreased, while CAV1 levels are normal. (B) Knockdown of CAV3 by siRNA diminishes insulin‐stimulated glucose uptake in HL‐1 cells. *P < 0.05 versus scrambled control, *t* test. (C) Insulin‐stimulated glucose uptake is decreased in palmitate‐fed *Cav1* null mice. Glucose uptake assays were performed on isolated cardiomycoytes from *Cav1* null mice after 12 weeks on standard, MCT, or palmitate diets. Palmitate diet group showed decreased insulin‐stimulated uptake. *n *= 4–10. Data are means ± SE, one‐way ANOVA, with Student–Newman–Keuls post hoc comparison.

HL‐1 cells are immortalized cell line and they were used for in vitro experiments due to challenges in maintaining primary cardiomyocyte cultures. The feasibility of CAV3 knockdown and specificity of *Cav3* siRNA were first verified by western blots. Figure 5A shows that 20 h transfection of HL‐1 cells with CAV3 siRNA caused an 80% decrease in Cav3 protein without any effect on CAV1 levels. Using this validated in vitro cellular model, we then addressed the role of CAV3 in insulin sensitivity. Figure 5B clearly demonstrates that insulin‐stimulated glucose uptake was absent in siRNA‐transfected HL‐1 cells compared to scrambled siRNA control cells, thus further supporting an important role of CAV3 in insulin sensitivity. To support these in vitro data with in vivo experiments, we used CAV1 null mice fed standard or high‐fat diets and isolated cardiomyocytes after 12 weeks to test their insulin‐dependent glucose uptake rates. The rationale for CAV1 null mice is that these mice only express CAV3 in their heart and we can thus exclude potential compensatory effects of caveolin isoform upregulation. Consistent with our other results, Figure 5C shows that PALM‐induced loss of CAV3 causes insulin resistance in isolated cardiomyocytes.

### Insulin‐independent activation of IR substrate‐1 (IRS‐1) and diminished Akt activation by palmitate

**Figure 6:**
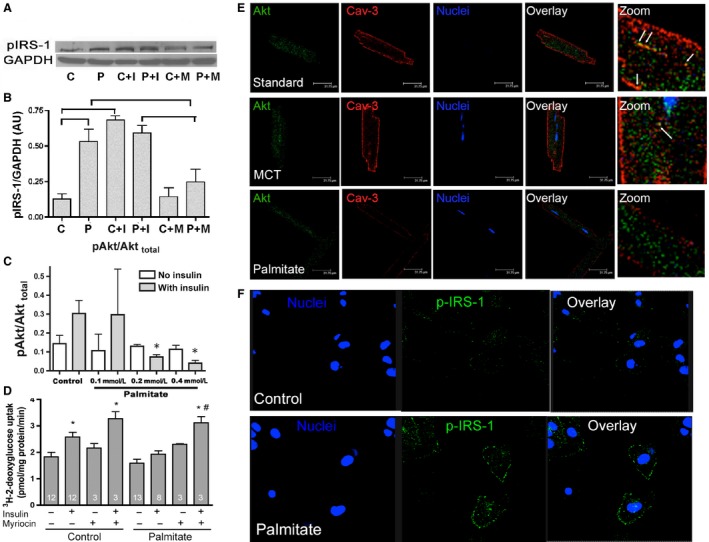
Insulin‐independent activation of IRS‐1 and decreased insulin‐stimulated pAkt and glucose uptake by palmitate. (A) Western blot analysis shows increased serine phosphorylation of IRS‐1 by 0.4 mmol/L palmitate in the absence of insulin, which is partially prevented by 40 nmol/L myriocin. Control cells showed increased IRS‐1 serine phosphorylation only following 10 min of 200 nmol/L insulin challenge. GAPDH was used as protein loading control. C: control, P: palmitate, C+I: control + insulin, P+I: palmitate + insulin, C+M: control + myriocin, P+M: palmitate + myriocin. (B) Quantification of (A), means ± SE,* n* = 3 with three replicates each. Brackets indicate *P* > 0.01, one‐way ANOVA, Newman–Keuls post hoc comparison. (C) The ratio of pAkt to total Akt was determined by western blotting in HL‐1 cells following 18 h treatment with control or varying concentrations of palmitate with and without 200 nmol/L insulin challenge for 10 min (as shown in gray and white bars, respectively). Palmitate decreases insulin‐dependent pAkt level. Data are means ± SE, one‐way ANOVA, Student–Newman–Keul post hoc comparison, *P < 0.05, *n* = 3. (D) 2‐deoxy‐d‐[^3^H]‐glucose uptake was determined in HL‐1 cells following 18 h treatment with control or 0.4 mmol/L palmitate medium in the absence or presence of insulin or myriocin. Insulin‐stimulated glucose uptake is decreased in palmitate‐treated cells, but treatment with myriocin (40 nmol/L) to maintain CAV3 levels normalizes insulin‐dependent glucose uptake. Data are means ± SE, one‐way ANOVA, Student–Newman–Keul post hoc comparison, *P < 0.01 versus control, ^#^ *P *< 0.01 versus palmitate. (E) Localization of Akt and CAV3 in confocal images of primary isolated mouse cardiomyocytes from mice fed standard, MCT, or palmitate diet for 12 weeks. Akt and CAV3 colocalize in cardiomyocytes from standard diet‐fed mice and this is maintained in mice fed MCT diet. However, in palmitate diet‐fed mice there is no colocalization mainly due to the loss of CAV3 from the t‐tubules system. Similar results obtained from three different isolations. (F) Representative confocal images of HL‐1 cells showing constitutive activation of IRS‐1 by palmitate. As suggested from (A) exposure to palmitate causes constitutive phosphorylation of IRS‐1 in HL‐1 cardiomyocytes with localization to the plasma membrane. Experiment repeated four times with similar results.

Previously, we have reported that palmitate diet feeding induces a loss of cardiac CAV3 in mice (Knowles et al. 2013). Therefore, to address the role of CAV3 in insulin signal transduction pathway, we determined the effects of palmitate‐induced loss of CAV3 on the activity of IR downstream targets, IRS‐1 and Akt. Representative western blots in HL‐1 cells (Fig. 6A) show that 18‐h exposure with palmitate (0.4 mmol/L) increases serine phosphorylation of IRS‐1 (pIRS‐1) even in the absence of insulin and it is comparable to the increased pIRS‐1 in control cells stimulated by 200 nmol/L insulin. Palmitate‐induced increase in pIRS‐I was partially reduced by 40 nmol/L myriocin, an inhibitor of serine palmitoyltransferase 1, that clearly links the insulin resistance to the production of sphingolipids from palmitate. Importantly, the constitutive activation of IRS‐1 in palmitate‐treated cells without insulin was also observed by confocal microscopy. Figure 6E shows HL‐1 cells with constitutive activation and localization of IRS‐1 to the plasma membrane in the presence of palmitate. In contrast, insulin‐stimulated increase in serine phosphorylation of Akt (pAkt) in HL‐1 cells was dose dependently inhibited by palmitate (Fig. 6C), with maximal inhibition observed at 0.4 mmol/L palmitate. Confocal immunofluorescence images (Fig. 6D) of primary isolated cardiomyocytes also suggest that Akt is in close proximity to caveolae in mice fed with standard or MCT diet, while this localization is lost in mice fed PALM diet for 12 weeks. Using the similar experimental conditions in HL‐1 cells, we then determined 2‐deoxy‐d‐[3H]‐glucose uptake in the absence or presence of insulin or myriocin. Figure 6C shows that palmitate treatment decreased insulin‐stimulated glucose uptake in HL‐1 cells compared to control condition; however, treatment with myriocin (40 nmol/L) enhanced insulin‐stimulated glucose uptake in both control and palmitate‐treated cells. Thus, these findings support a critical role of CAV3 in the regulation of insulin signal transduction cascades particularly in a high‐fat environment.

### Comparison of caveolin proteins, insulin receptor cascade components, and CD36 expression in cardiac muscle

**Figure 7:**
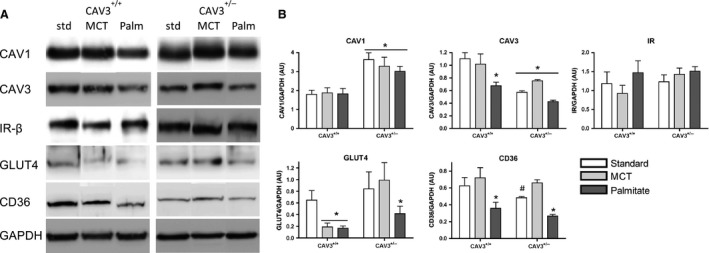
Effect of high‐fat feeding on the expression of CAV1 and 3, IR, IRS‐1, GLUT4, and CD36 in hearts (A, B) of littermate CAV3^+/+^ and CAV3^+/−^ mice. Expression of CAV1 is increased in hearts of CAV3^+/−^ mice compared to CAV3^+/+^ hearts, and the expression level is independent of lipid exposure. In contrast, CAV3 expression is reduced in CAV3^+/−^ mice compared to CAV3^+/+^ mice and is further decreased with exposure to palmitate in both groups of mice. The insulin receptor expression is not different between hearts of CAV3^+/+^ and CAV3^+/−^ mice and is not changed by dietary lipid exposure. GLUT4 levels decreased with MCT and palmitate exposure in CAV3^+/+^ mice, whereas in CAV3^+/−^ mice only palmitate caused a significant drop in GLUT4 expression. CD36 is markedly decreased by palmitate in CAV3^+/+^ hearts and also in CAV3^+/−^ mice on standard diet. Lipid diets had a differential effect on CD36 in CAV3^+/−^ mice, with MCT diet increasing expression and palmitate decreasing expression. Western blotting was repeated on a total of 6–8 samples with similar results. Data are means ± SE, *P < 0.05 versus CAV3^+/+^ STD, two‐way ANOVA, Bonferroni–Dunn post hoc comparison. ^#^ *P *= 0.056 for CD36 levels in CAV3^+/−^ hearts versus CAV3^+/+^ hearts.

The effect of dietary lipids on the expression of CAV1, CAV3, IR, GLUT4, and CD36 in cardiac muscle is shown in Figure 7. In wild‐type CAV3^+/+^ hearts there is no dietary lipid effect on the expression of CAV1. Surprisingly, the CAV3^+/−^ mice show upregulation of CAV1 in the heart (Fig. 7A and B). This is an unexpected finding because until now, a compensatory effect between the different caveolins has not been demonstrated. However, these data are from whole heart tissue extracts, thus an upregulation of CAV1 in cardiomyocytes remains to be shown. As expected, CAV3 levels are ~50% lower in CAV3^+/−^ mice and are further diminished by palmitate diet in both CAV3^+/+^ and CAV3^+/−^ hearts as well as in skeletal muscle. Insulin receptor levels in the heart and skeletal muscle are the same in CAV3^+/+^ and CAV3^+/−^ mice and remain unchanged by lipid diets. GLUT4 levels are equal in the hearts of CAV3^+/+^ and CAV3^+/−^ mice fed STD diet and decreased in CAV3^+/+^ hearts of animals fed MCT and palmitate diets, whereas in CAV3^+/−^ mice GLUT4 levels only decrease in palmitate diet‐fed mice. Most interestingly, CD36 levels decrease in heart of CAV3^+/−^ mice compared to CAV3^+/+^ mice, suggesting that they are linked to the expression of CAV3. Palmitate diet decreased CD36 expression further in both CAV3^+/+^ and CAV3^+/−^ hearts. MCT diet prevented a drop in CD36 expression, and even in the CAV3^+/−^ mice seemed to have a pronounced protective effect on CD36 levels, which were similar to those in CAV3^+/+^ hearts.

## Discussion

The goal of this study was to determine whether partial loss of CAV3 increases the susceptibility to lipid‐induced glucose intolerance and to define the underlying mechanisms. We used a novel CAV3 heterozygous (CAV3^+/−^) mouse model without any overt skeletal or cardiac muscle phenotype, in which expression of total CAV3 protein is ~twofold lower compared to litter mate wild‐type (CAV3^+/+^) mice. We found that lower expression levels of CAV3 accelerate the time of palmitate‐induced glucose intolerance by 50%, with mild changes of in vivo cardiac contractile function. Further investigation of the insulin receptor cascade showed normal IR expression levels in all groups, but constitutive activation of IRS‐1 and diminished Akt activation and lower glucose uptake under all investigated mechanisms of loss of function of CAV3. In addition, we found a role of CAV3 in cardiac CD36 expression. This suggests that CAV3 is an important regulator of not only the insulin receptor in the mouse heart, but also of the long‐chain lipid translocase, CD36.

### IR shares a membrane microdomain with CAV3 in cardiomyocytes

Direct interaction of CAV with the IR remains controversial. Using CAV3 null mice, it was shown in skeletal muscle that the stability of the IR and its signaling is dependent on CAV3/caveolae expression (Oshikawa et al. 2004; Capozza 2005), however, a direct physical CAV3–IR interaction was not addressed. Fecchi et al. (Fecchi et al. 2006) demonstrated by coimmunoprecipitation that the IR localizes to caveolae in myocytes. In contrast, Souto et al. (Souto et al. 2003) used immunoelectron microscopy to show that IR was not within caveolae in adipocytes and did not coimmunoprecipitate with CAV1. Recent structural and bioinformatics analysis of caveolins and the caveolin‐binding motif (Collins et al. 2012) argues against a direct physical interaction between caveolins and IR. This conclusion was based on the structural analysis of the caveolin CSD domain, which is in very close contact with the inner membrane leaflet, thus potentially preventing its involvement in protein–protein interactions. Additionally, caveolins and caveolae are in some studies considered signaling inhibitors (Okamoto et al. 1998). However, studies performed in transgenic mouse models have demonstrated that insulin signaling may not show caveolin‐mediated inhibition, but instead might require CAV1 or CAV3 for functioning (Okamoto et al. 1998; Yamamoto et al. 1998). Our co‐IP and FRET data in HL‐1 cardiomyocytes provide further support that the IR and CAV3 are in close proximity. The colocalization of Akt and CAV3 further supports our conclusion that CAV3 and the insulin receptor cascade components are in close proximity in cardiomyocytes. Also, the knockdown of CAV3 by siRNA shows clearly that CAV3 is important in regulating glucose uptake by cardiomyocytes. Thus, these findings from different experimental conditions suggest that IR signaling shares a membrane microdomain with CAV3 in cardiomyocytes and that high‐fat feeding conditions can effectively disrupt this membrane association.

### High‐palmitate diet, CAV3, insulin resistance, and cardiac function

It is now well established that increased saturated dietary fat plays a key role in promoting loss of insulin sensitivity and thus insulin resistance (Riccardi et al. 2004). CAV3 and IR interactions and the effects on insulin signaling have been characterized in skeletal muscle (Oshikawa et al. 2004; Otsu et al. 2010); however, the role of CAV3 in cardiac insulin signaling is poorly defined, especially the underlying mechanisms of high fat‐induced insulin resistance are still unclear. Insulin resistance, defined as reduced responsiveness of tissues to normal insulin concentrations, is a principal feature of type 2 diabetes (T2DM), and leads to compensatory hyperinsulinemia. It is also frequently associated with a number of metabolic abnormalities including obesity, hyperglycemia, and dyslipidemia. The enhanced susceptibility of the CAV3^+/−^ mice to palmitate‐induced glucose intolerance suggests that CAV3 protein levels are important in the regulation of cardiac glucose metabolism. It further suggests that about 50% of CAV3 expression is sufficient to maintain normal insulin receptor function and normal contractile function in the heart. Further decrease in CAV3 expression induced by dietary lipid stress causes CAV3 content to fall under 50% of normal level when normal glucose metabolism cannot be maintained.

To further define the role of CAV3 and its loss on insulin signaling pathway at a molecular level, we determined the effects of palmitate‐induced loss of CAV3 on the expression level of the IR and on the activity of IRS‐1 and Akt and expression of GLUT4, which are downstream of the IR. Palmitate exposure did not change expression levels of the IR, but increased serine phosphorylation of IRS‐1 with enhanced localization to the plasma membrane (Fig. 6A and E) and decreased insulin‐stimulated serine phosphorylation of Akt (Fig. 6B and D). GLUT4 levels in the heart decreased in CAV3^+/+^ and CAV3^+/−^ mice during palmitate feeding (Fig. 7A and B) with associated lower glucose uptake rates (Fig. 5C). To determine whether the changes in glucose metabolism are caused by a shift to the preferred lipid substrate or more indirectly through palmitate metabolism, we used myriocin, an inhibitor of serine palmitoyltransferase 1 (SPT1), which catalyzes the first step of ceramide synthesis from palmitate. The rescue of insulin sensitivity in HL‐1 cells (Fig. 6C) and the normalization of IRS‐1 phosphorylation (Fig. 6A) strongly suggests that the effect of palmitate is mediated through the accumulation of sphingolipids in the membrane, as we have previously shown (Knowles et al. 2013). The IR, IRS‐1, Akt, and GLUT4 data further suggest that loss of CAV3 dissociates signaling through the insulin receptor cascade, because enhanced IRS‐1 activation does not lead to increased Akt activation.

### Lipid‐induced loss of CD36

CD36 is the most important translocase for long‐chain unesterified fatty acids in the heart (Glatz et al. 2010). Souto et al. (Souto et al. 2003) have demonstrated that CD36 localizes to caveolae in adipocytes. Mutations in CD36 in humans lead to an increased risk of diabetes‐associated cardiovascular disease and metabolic syndrome (Ma et al. 2004; Love‐Gregory et al. 2008). Recently, Pietka et al. (Pietka et al. 2012) demonstrated that CD36 null mice have prolonged calcium transients in the fasting state and increased incidents of sudden death. Our findings of lower CD36 levels in mouse hearts with lower levels of CAV3 (CAV3^+/−^ and palmitate diet‐fed groups) suggest that CD36 and CAV3 interact in the heart as well. In addition, the hypercontractile phenotype of the 6‐week‐old CAV3^+/−^ mice on standard laboratory chow can be explained by increased intracellular calcium levels. At the 6‐ and 12‐week time points the additional stress of the palmitate diet combined with the inability to appropriately use lipids as substrates, due to loss of CD36, or glucose, due to impaired insulin sensitivity, potentially contributes to these hearts being energetically challenged and likely causes the decrease in contractile function that we observed by echocardiography. The same is seen in the CAV3^+/+^ hearts that at 12 weeks of palmitate feeding lose CD36 expression and insulin sensitivity, and show a pronounced slowing in diastolic velocity.

In conclusion, the present study with a novel animal model provides direct evidence that partial loss of CAV3 protein, independent of any cardiac phenotype, renders mice more susceptible to high lipid‐induced glucose intolerance associated with a mild cardiac contractile dysfunction at 12 weeks of feeding. This dysfunction is in part not only due to the dysregulated IR cascade, but also due to the concomitant loss of CD36 and decreased lipid uptake into the heart.

## Conflict of Interest

None declared.
